# Eveningness and preeclampsia in gestational diabetes – a response to the letter “Chronotype of pregnant women with diabetes mellitus: what is the relationship with maternal and fetal outcomes”

**DOI:** 10.20945/2359-4292-2023-0141

**Published:** 2024-02-02

**Authors:** Cristina Figueiredo Sampaio Facanha, Victória Sudário Alencar, Paula Soares Machado, Rejane Belchior Lima Macêdo, Pedro Felipe Carvalhedo de Bruin, Adriana Costa e Forti, Thaine Mirla Rocha, Veralice Meireles Sales de Bruin

**Affiliations:** 1 Universidade Federal do Ceará Departamento de Medicina Fortaleza CE Brasil Departamento de Medicina, Universidade Federal do Ceará, Fortaleza, CE, Brasil; 2 Centro Universitário Christus Departamento de Medicina Fortaleza CE Brasil Departamento de Medicina, Centro Universitário Christus, Fortaleza, CE, Brasil; 3 Centro Integrado de Diabetes e Hipertensão do Ceará Secretaria Estadual de Saúde do Ceará Fortaleza CE Brasil Centro Integrado de Diabetes e Hipertensão do Ceará (CIDH) – Secretaria Estadual de Saúde do Ceará, Fortaleza, CE, Brasil

**DEAR EDITORS AND COLLEAGUES,**

Thank you for your interest and comments on our study. This work evaluates differences in women with gestational diabetes mellitus (GDM) regarding the presence of morningness/eveningness or identified chronotypes investigated during pregnancy in a robust cohort of 305 GDM patients. Our results show that GDM women with evening preference are more likely to develop pre-eclampsia (PE), their offspring were more likely to be admitted on neonatal intensive care unit and there was a marginal increased risk of prematurity (1).

Gestational diabetes mellitus (GDM) is independently associated with PE in singleton pregnancy (2), and important factors as body mass index (BMI), arterial hypertension and blood glucose are closely related with the occurrence of PE in GDM (2-4). Other factors such as older maternal age, parity, gestational weight gain, OGTT blood glucose levels, and even type of treatment have also been suggested, however, the influence of these factors are controversial (2).

Among other risk factors related to PE in the general population, nulliparity was found in 24.8% of the patients, and it was not related with eveningness (p = 0.18) nor PE (p = 0.30). The prevalence of smoking during pregnancy was very low, counting for less than 2% (5 patients). For this reason, it could not be included in the present analysis due to perfect separation error. Regarding the influence of age, typically, older age is associated with adverse pregnancy outcomes and PE. As expected, eveningness was associated with younger age in this group (5), and despite this counterintuitive fact eveningness remained associated with PE in GDM patients. Arterial hypertension and obesity, which are the most common comorbidity in GDM (6) were equally present, independent of chronotype. Other minor factors were also examined and were not influential for eveningness, preeclampsia nor other neonatal variables, which led to their non-inclusion in the multivariate model.

Considering additional concern regarding the influence of multiple factors on the outcome of pre-eclampsia in GDM, we performed a multivariate logistic regression analysis including relevant clinical variables. Lower levels on the Horne-Östberg Morning-Evening Questionnaire, which reveals evening preference (7), remained associated with PE After controlling for fasting blood glucose, BMI, age and arterial hypertension (Enter Model) (OR: 0.94; CI: 0.90-0.98; p < 0.005) (Table 1).

**Table 1:** Logistic regression multivariate analysis of variables influencing pre-eclampsia (enter model)

Variables	OR [CI]	p-value
MEQ	0.94 [0.90-0.98]	<0.005**
HBP	11.7 [4.49-30.9]	<0.005**
FBG	1.00 [0.97-1.03]	0.58
BMI	0.96 [0.88-1.04]	0.39
Age	0.94 [0.87-1.02]	0.16

MEQ: Morningness Eveningness Questionnaire; HBP: arterial hypertension; FBG: fasting blood glucose; BMI: body mass index.

** p<0.01.

In the original manuscript, we performed a multivariable logistic regression analysis to examine the relationship of independent variables to PE outcome. Previously, we defined that only factors with p < 0.2 should be considered in the analysis. However, as an additional care, reviewers insisted that age should be included, as it is an important determinant to eveningness behavior. After analysis, age was non-influential to the results.

Pre-eclampsia is a common complication of GDM pregnancy. The development of a clinical model to assess the risk of PE is important. We suggest that future studies should include eveningness in the model based on clinical data to predict the development of PE in women with GDM.
